# Beam-steering of a single-element nano-antenna with two transferring dielectric flat lenses

**DOI:** 10.1038/s41598-025-03482-x

**Published:** 2025-05-26

**Authors:** Fatma E. Helmy, Ibrahim I. Ibrahim, Amany M. Saleh

**Affiliations:** Electronics and Communications Department, Faculty of Engineering, Helwan University, Cairo, 11795 Egypt

**Keywords:** Hybrid plasmonic optical nano-antenna, Switched-beam nano-antenna, Dielectric flat lens nano-antenna, Lens antennas, Transferring lens, Engineering, Nanoscience and technology, Optics and photonics

## Abstract

A novel idea for mechanical beam-steering based on a single hybrid plasmonic nano-antenna (NA) with transferring lenses is presented in this research. Two inhomogeneous rectangular dielectric flat lenses modelled with different materials are used in the switched-beam nano-antenna (NA) to boost and steer radiation in a specific direction by shifting each lens. Firstly, a hybrid plasmonic NA design with two movable lenses is shown. Electromagnetic full-wave simulations examine the performance of this device, and the complete system is numerically evaluated. A conventional elliptical patch NA without lenses produces a gain of up to 10.7 dBi and a return loss of − 14.41 dB. The design with two gradient-index dielectric flat lenses is introduced to enhance antenna performance by improving gain while minimizing beam width. Furthermore, the beam-steering capabilities by displacement of the two lenses according to different positions of lenses along the X and Y-direction. By using the two gradient-index dielectric flat lenses, the gain is increased to 20.3 dBi with an improvement in the return loss reach to -24.9 dB compared with traditional NA. In addition, the beam-steering capabilities were achieved with a range  ± 45° × ± 40° with acceptable average antenna gain, side-lobe levels, and half power beam-width of 18 dBi, − 7.4 dB and 10.7° respectively. Moreover, the PSO algorithm is provided to maximize the pre-specified fitness function.

## Introduction

Optical wireless communication (OWC) offers several benefits, including high communication capacity, robust security, concentrated power, and easy networking. Due to their extraordinary qualities, optical nano-antennas (NA) have garnered much interest. Optical nano-antennas are devices used to regulate light in micro and nanometer dimensions^1–7^. There are several applications for these light-controlling properties, including light trapping in solar cells^8,9^, plasmonic biosensors^10^, sub-wavelength imaging devices^11–13^, and optical wireless communication systems^14–17^. More flexibility in the applications above can be made possible by dynamically controlling the radiation pattern of nano-antennas or beam-steering capabilities. This is especially useful for applications like light detection and ranging (LIDARs)^18,19^, optical communication^16^, authentication^20^, holography^21^, and imaging^22^. At the core of optical wireless-broadcasting communication connections, beam-steering devices are crucial for data exchange and allocation. Large steering angles, arbitrary channel counts, reconfigurability, and ultracompactness are characteristics of the perfect beam-steering system. Optical wireless-broadcasting communication techniques will be significantly restricted because these requirements have only been partially met by conventional beam-steering devices based on waveguides, micro-electrical mechanical systems, spatial light modulators, and gratings^3^. Many techniques have been proposed to provide optical beam-steering, including leaky wave antennas^23–26^, metasurfaces with programmable unit cells^27–33^, and phased array antennas^34–36^. All of the previously recognized systems, however, have disadvantages and limitations of their own, necessitating a continuous research stream focused on developing new strategies and tactics for optical beam-steering.

Leaky wave structures are utilized for decreasing size and eliminating the requirement for phase shifters. Other limitations of leaky wave antennas include a limited field of view (FOV) and significant loss^23–26^. These structures can be classified into single and multi-tone groups. The beam rotation in multi-tone leaky wave antennas is accomplished by changing the radiation wavelength, which necessitates the use of expensive and high-bandwidth lasers^23,24^. Single-tone structures, on the other hand, operate by varying the refractive index throughout a single wavelength. This method largely modifies the refractive index thermally, making it a low-speed technique^25,26^.

Using adjustable metasurfaces is an additional method of steering radiated beams^27–34^. Metasurfaces are a two-dimensional variant of metamaterials made up of a series of nano-antennas, each of which provides a certain reflected amplitude or phase. Metasurfaces can be constructed with tunable materials, such as vanadium dioxide (VO2)^27,28^, indium tin oxide (ITO)^29,30^, and phase-change materials (PCMs)^31,32^ such that their response can be controlled dynamically Wide FOV, relatively high-speed steering, and a narrow radiation beam are all possible with tunable metasurfaces utilized for beam-steering. However, the complexity and cost of these structures rise since each unit cell employed in the production of metasurfaces needs to be calibrated individually. To address this issue, lens-based devices which operate in the optical^37–43^ and microwave^44–46^ regimes were developed.

An alternative strategy is to use phased array antennas, which are frequently employed in the microwave range to facilitate beam scanning^34–36^. These antennas consist of a series of identical optical nano-antennas, each of which has an adjustable phase shifter attached to regulate the beam. Wide beam scanning, excellent resolution, and narrow beam width are among the benefits of optical phased array antennas. However, their possible uses are limited by several drawbacks and limitations, including their size, slowly adjusting phase shifters, and high lobe damage rate.

As mentioned in^47–56^, several types of metamaterial (gradient-index) lenses have been developed. Phase shifters are not necessary for integrated structures that have a Luneburg lens^47–49^ or a Rotman lens^50^, which allows beam-steering over a large scanning range. Luneburg lenses are gradient-index lenses with a spherical or hemispherical shape that can be used in a single-beam scanning plane. In^51^, Fresnel zone plate lenses are utilized to alter the feed antenna’s phase, resulting in a naturally narrowband conception at specified locations.

In^52^, inhomogeneous gradient-index dielectric flat lens design and numerical simulation in the optical range were provided for wireless optical communication. The performance of this lens in terms of overall performance, beam-scanning capability, maximum possible gain, and bandwidth efficiency. A two-lens scan can be positioned significantly closer to the source array enhancer than a single-lens device, as shown in^53^. A demonstration of beam-steering techniques at 1550 nm is presented in^54^ with an 8 × 8 hybrid plasmonic NA array. By employing a deep neural network (DNN) with or without a lens to forecast the correct feeding phases of the 64 elements, the beam is steered in the phased array antenna. In [55], an integrated optical system consisting of five switchable NAs and a reflecting meta-lens is connected to provide optical beam-steering at the standard 1550 nm wavelength for telecommunication. Unfortunately, their fabrication complexity and loss are substantial. Continuous beam-steering with low power consumption, as shown in^56^, is highly desired in contemporary antenna designs. A basic mechanical technique is demonstrated for accomplishing beam-steering in the Fabry–Perot resonator antenna (FPRA). The FPRA has a gain of 14.9 dBi and can continuously guide its beam in the azimuthal plane at around 50°.

To address the issues described above, we introduce in this research beam-steering of a single plasmonic NA element with an elliptical patch created in^57^ utilizing the new idea of two transferable rectangular dielectric flat lenses for optical wireless applications at the conventional telecommunication wavelength of 1550 nm (193.5 THz). The entire structure is numerically analyzed, and its performance is studied using electromagnetic full-wave simulations. The simulation results demonstrate various advantages for the developed structure, as compared to previously reported works. Consequently, The PSO is then used to optimize the position of the two lenses to steer the radiation pattern in a certain direction at the common telecom wavelength of 1550 nm (193.5 THz). Several engineering applications have effectively used the PSO technique as a vital tool for finding optimal solutions.

The paper that has been considered is structured as follows: “Design considerations and simulation methodology section” presents the design considerations and simulation methodology of the plasmonic single NA with two transferring dielectric flat lenses, along with a comparative analysis with the results of earlier publications. “Simulated results and discussion” section presents and discusses the findings. The results are finally summarized in “Conclusion” section.

## Design considerations and simulation methodology

**Fig. 1:**
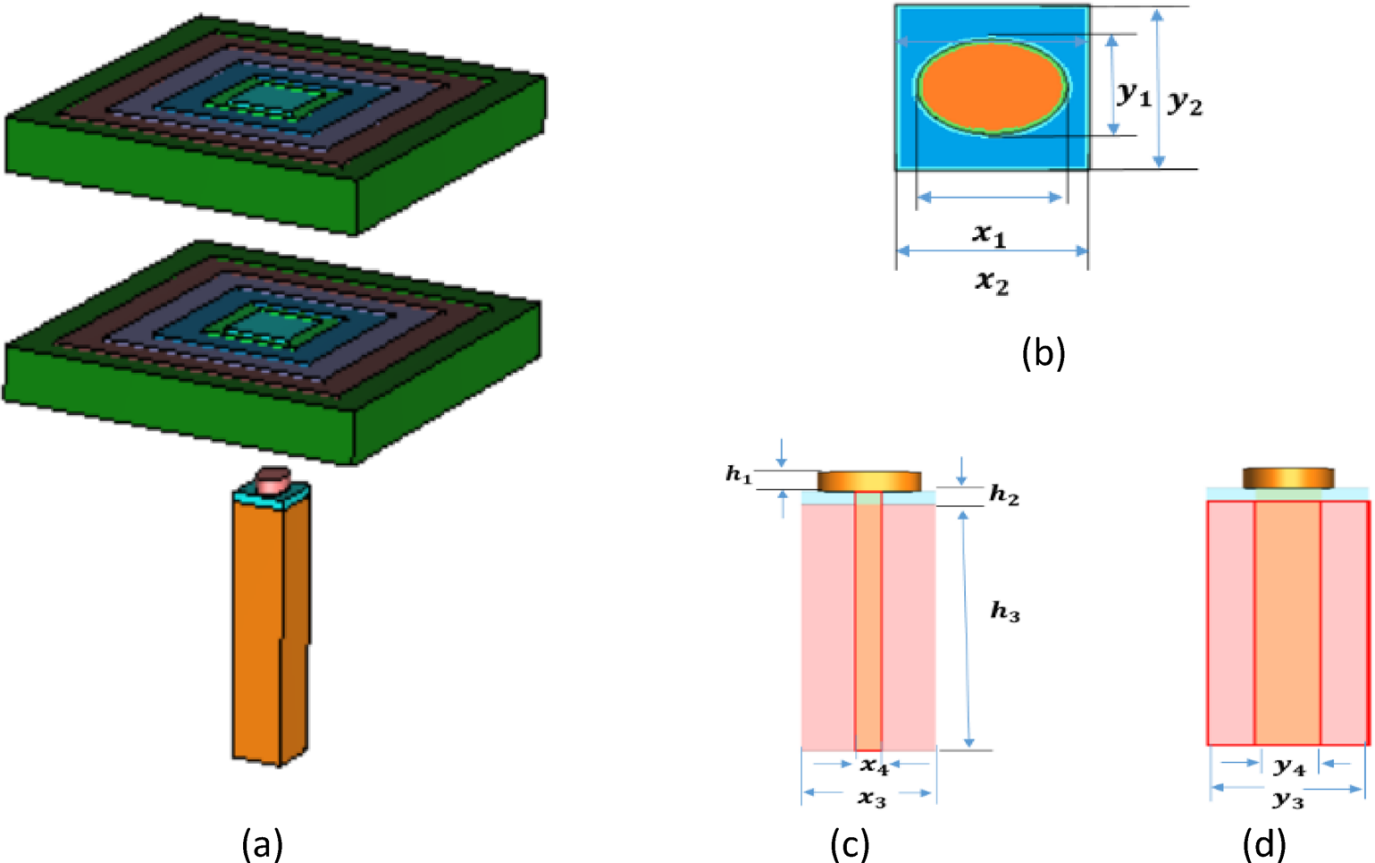
(**a**) 3D schematic illustration of the produced plasmonic NA with two transferring rectangular dielectric flat lenses, (**b**) Top view of the elliptic patch plasmonic NA fed by a silicon waveguide^58^, (**c**) Front view of NA, and (**d**) Side view of NA.

Figure 1a shows an illustration of the introduced plasmonic NA with two transferring rectangular dielectric flat lenses along ± x, ± y directions to steer the beem for optical wireless applications at the conventional telecommunication wavelength of 1550 nm (193.5 THz). Electromagnetic full-wave simulations are used to study the structure’s performance and perform a numerical analysis. The operating principles and design approach for the antenna are detailed below.

### The geometrical structures of the plasmonic NA

The schematic diagram of the plasmonic NA we introduced in^57^ is shown in Fig. 1b, c, and d. The elliptic silicon (Si) patch block, the silver (Ag) block, and the silicon waveguide coated with silicon dioxide (SiO_2_) make up the plasmonic NA. The length of the edge along the x- and y-axes determines the elliptic block’s width and length. As shown in Fig. 1b, c, the width, length, and height of the silicon patch block are represented by the parameters (x_1_ (major axis), y_1_ (minor axis), and h_1_), which are equivalent to (850, 625, and 300) nm, accordingly. Likewise, the parameters (x_2_, y_2_, and h_2_) representing (1100, 1100, 200) nm, respectively, indicate the width, length, and height of the silver block. Similarly, the parameters (x_3_, y_3_, and h_3_) denote the silicon dioxide coating’s width, length, and height, which are, respectively, equivalent to (1100, 1100, and 8680) nm. The dimensions of (x_4_, y_4_) in Fig. 1c, d indicate the silicon waveguide lengths along the x- and y-axes, and they equate to (220, 450) nm. Silver’s relative dielectric constant is − 129 + j3.28^58^. The relative dielectric constants of silicon patch and silicon dioxide are 12.11 and 2.1, respectively. Light is fed into the plasmonic NA through the silicon waveguide incorporated in the silicon dioxide coating, which is coupled to the silicon patch block through the silver block. From the bottom of the silicon waveguide, light with transverse electric polarization, or x polarization, is fed and released upward and vertically. The following formulas establish the lower cutoff frequency for a specific mode in a rectangular waveguide:

1 $$\:{\left({f}_{c}\right)}_{mn}=\frac{c}{2}\sqrt{{\left(\frac{m}{a}\right)}^{2}+{\left(\frac{n}{b}\right)}^{2}}$$

where the waveguide’s width and length are indicated by the letters a and b. ‘m’ indicates the lowest cut-off frequency of the dominant mode in a given waveguide, whereas ‘n’ denotes the range of potential modes.

### Dielectric flat gradient-index lens design

**Fig. 2:**
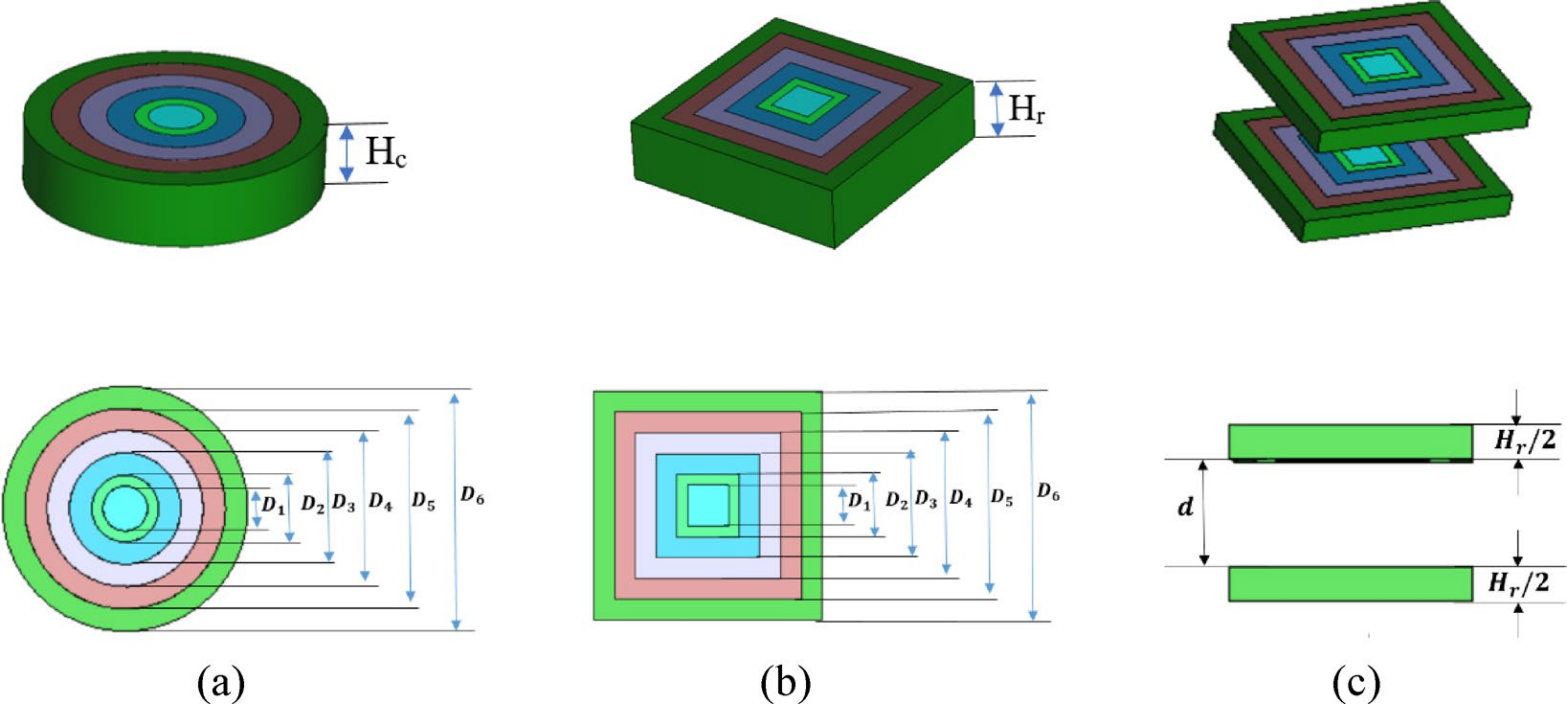
Dielectric flat gradient-index lens structure. (**a**) circular lens, (**b**) rectangular lens, (**c**) two transferring lenses.

An optimized inhomogeneous dielectric flat lens composed of various materials is utilized to direct and intensify the radiation in a certain direction^52,60^. The lens was designed with six concentric rings constructed of varying permittivity materials (ε_r_) to improve the radiation pattern when lighted from its focal position. Since the feeding point is altered along the x or y-axis, the differing permittivity values result in a linear phase slope that purely directs the beam along the gradient-index axis. The following materials’ permittivity was determined in the surrounding rings of the introduced circle lens: $\:{\varepsilon\:}_{{r}_{1}}$ > $\:{\varepsilon\:}_{{r}_{2}}$ > $\:{\varepsilon\:}_{{r}_{3}}$ > $\:{\varepsilon\:}_{{r}_{4}}$> $\:{\varepsilon\:}_{{r}_{5}}$ > $\:{\varepsilon\:}_{{r}_{6}}$ where the maximum permittivity (ε_r1_) at the center of the lens and a continuous decrease to the minimum permittivity $\:{\varepsilon\:}_{{r}_{6}}$ on the outside ring with values of 7.1, 6.79, 6.01, 4.99, 3.92, and 2.9. Eqs. (2) and (3) offer the design and construction of lenses with consistent thickness for a flat form. It is achievable to determine the radii (R_i_) for each dielectric zone and the lens thickness H, which is proportional to the two adjacent permittivities at the lens, by using^51,61^:

2 $$\:{R}_{i}=\sqrt{2Fi\left(\frac{{\uplambda\:}}{P}\right)+{\left(i\frac{{\uplambda\:}}{P}\right)}^{2}} \quad i=2,\:3,\:\dots,\:P$$

3 $$\:H=\frac{{\uplambda\:}}{P(\sqrt{{\epsilon\:}_{ri}}-\sqrt{{\epsilon\:}_{ri-1}}} \quad i=2,\:3,\:\dots,\:P$$

where P indicates the phase correcting index and its value is 6, F depicts the focal length, and the design wavelength is represented by λ. As shown in Fig. 2a, the produced dielectric flat gradient-index lenses’ diameter (D_1_, D_2_, D_3_, D_4_, D_5_, D_6_), which are equal to the numbers (1409, 2113, 3522, 4931, 6340, and 7750), respectively^57^. The focal length F and thickness H_c_ of the introduced lens with values of 1937.5 nm (D/4) and 2170 nm (1.4λ), respectively. Concerning the circular lens, Fig. 2b illustrates the creation of a rectangle lens whose dimensions match the diameters of the lenses. Additionally, the thickness of the newly rectangle lens H_r_ is equivalent to the circular lens’s H_c_. The proposed two lenses, as illustrated in Fig. 2c, have the same dimensions as the rectangular lens except the height is reduced to half the height of the rectangular lens as shown in Fig. 2c. For the two-lens system, the angle scan enhancement factor α is given by^53,62^:

4 $$\:\alpha\:\equiv\:1-\frac{\text{d}}{{F}_{s}},$$

where d is the distance between the two lenses and F_s_ is the focal length of the second lens. For the two lenses to function as a scan-improving device, the lens and geometry characteristics must meet d − F − F_s_ = 0, where F is the focal length of the first lens. By optimizing d between the two lenses, the value will be 4570 nm. The lens concentrates incident light to a specific point by behaving as a kind of passive phase shifter. This lens exhibits two important properties when used with antennas: concentrated signal power directed to a particular sub-region of the antenna array and concentrated signal power towards the front end, which results in high gain and directivity^54^. Because of these characteristics, the lens can be used to create optical beamforming systems in a useful manner.

### Beam-steering of the two transferring lens

It is necessary to compute the gain to examine the developed NAs’ performance. Gain refers to the amount of power transferred from an isotropic source towards its direction of peak radiation. An antenna’s gain is the ratio of power applied to the antenna to the actual power emitted which can be computed using^59^:

5 $$G=\frac{U\left(\theta\:,\phi\:\right)}{{P}_{in}/4\pi\:},$$

where P_in_ represents the amount of electrical power the antenna receives from the transmitter and U(θ, φ) denotes the radiation intensity.

Devices that steer beams are essential for data allocation and exchange. To enable beam-steering, a new mechanical approach is presented for the transferring of two rectangular dielectric flat gradient-index lenses. The mechanical process produces a slower scanning speed. However, it decreases complexity and uses less energy to maintain mechanical motion. As a result, the mechanical technique represents a promising solution for beam-steering. The incident and the refracted waves would correspond to the generalized Snell’s law^63^. The phase gradient and beam tilt angle can be related, assuming a constant rate of phase change and an incident angle of 0° in the following way:

6 $$\frac{d\phi\:}{dx}=\frac{2\pi\:}{\lambda\:}\text{sin}\theta\:\:,$$

where dφ/dx denotes the phase gradient, λ represents the operating wavelength, and θ defines the beam tilt angle^56,64^. Mechanically adjusting one or both lenses to vary the aperture phase distribution, allowing the beam to be continually guided in the azimuthal plane while maintaining a broad angle.

The new idea is displacement of the two lenses in different directions to achieve maximum gain in the desired direction as mechanical beam-steering. While electronic technologies provide higher speeds and flexibility, mechanically controlled dielectric flat lens systems can be more cost-effective, energy-efficient, and robustness. The decision is based on the application’s particular requirements and limits:

- Simplicity and Energy Efficiency: Mechanical systems are generally simpler in design and operation. They do not require complex electronic control circuits, which can make them more energy-efficient and easier to maintain.
- Cost-Effectiveness: The manufacturing and operational costs of mechanical systems can be lower, as they do not need expensive electronic components or intricate fabrication processes.
- Robustness and Durability: Mechanical systems can be more robust and durable, as they are less susceptible to electronic failures and can operate in a wider range of environmental conditions.

Mechanically controlled dielectric flat lens systems are well-suited for applications where speed is not the primary concern but where reliability, energy efficiency, and cost-effectiveness are important. These systems can provide a robust and durable solution for a variety of applications as: low-speed communication systems, environmental monitoring, fixed surveillance systems, astronomical observations, industrial automation for tasks like material inspection or robotic guidance where beam-steering speed is not a primary concern, and medical applications due to their specific advantages, precision, and reduced electromagnetic interference.

As a comparison between mechanically controlled dielectric flat lens systems and electronic beam-steering methods, particularly in terms of speed, scalability, and real-world application potential. Mechanical systems are inherently slower because they rely on physical movement to steer the beam. This can limit their use in applications requiring rapid adjustments, such as LIDAR or high-speed optical communications. Furthermore, scaling mechanical systems can be challenging due to the increased complexity and potential for mechanical wear and tear. However, Mechanical Beam-Steering systems are often simpler and more energy-efficient, making them suitable for applications where speed is not critical. They can be used in scenarios where cost and energy consumption are more important than rapid beam adjustments. Therefore, while electronic methods offer superior speed and flexibility, mechanically controlled dielectric flat lens systems can be advantageous in terms of simplicity, cost, energy efficiency, and robustness. The choice depends on the specific needs and constraints of the application^65–67^:

1. Low-Speed Communication Systems: Ideal for communication networks where rapid beam-steering isn’t essential, these systems are simple and energy-efficient, helping to lower operational and maintenance costs.
2. Environmental Monitoring: Suitable for weather monitoring or environmental sensing, where beam-steering speed is less critical. Mechanical systems offer reliable, consistent performance over long periods, even in harsh conditions.
3. Fixed Surveillance Systems: Perfect for surveillance systems that don’t need rapid field-of-view changes. Their robustness and durability ensure long-term reliability and reduced maintenance.
4. Astronomical Observations: Used in telescopes and other astronomical instruments requiring precise but slow adjustments. Mechanical beam-steering systems provide the necessary precision without the complexity and cost of electronic systems.
5. Industrial Automation: Beneficial in industrial settings for tasks like material inspection or robotic guidance, where beam-steering speed isn’t a primary concern. These systems are cost-effective and energy-efficient, reducing overall operational costs.
6. Certain Medical Applications: Mechanical systems offer specific advantages in various medical fields:
  Medical Imaging: Used in systems like ultrasound or MRI, where precise but not necessarily rapid beam-steering is needed. Their robustness and reliability ensure consistent performance, crucial for accurate imaging.
  Radiation Therapy: Employed in targeted radiation therapy for cancer treatment, where beams must be precisely directed at tumor sites. The high precision and stability of mechanical systems help accurately target tumors while minimizing damage to surrounding healthy tissues.
  Surgical Navigation: Assist in guiding surgical instruments during procedures. The simplicity and energy efficiency of mechanical systems are beneficial in portable or battery-operated surgical navigation tools.
  Wearable Health Monitors: Used in devices that monitor vital signs or other health metrics, focusing on specific body parts. Their low power consumption and durability make them ideal for long-term use in wearable devices.
  Telemedicine: Utilized in remote diagnostic tools that require beam-steering to focus on different body parts. The cost-effectiveness and ease of maintenance of mechanical systems make telemedicine tools more accessible and reliable.

Therefore, mechanically controlled dielectric flat lens systems offer advantages in terms of precision, reliability, energy efficiency, electromagnetic interference reduction and cost-effectiveness, making them suitable for various applications where rapid beam-steering is not critical. However, each technology has its own set of advantages and trade-offs.

In this paper, particle swarm optimization (PSO) is used to maximize the gain of the produced NA due to the low computational complexity, convergence speed, high performance, and ability to seek the global optimum. PSO is frequently chosen over other optimization algorithms like Genetic Algorithms (GA) and Gradient Descent (GD) for beam-steering antennas due to several unique advantages^68–71^:

1. Convergence Speed: PSO typically converges faster than GA because it uses a simpler mechanism of updating positions and velocities of particles based on their own experience and that of their neighbors. GA involve more complex operations like selection, crossover, and mutation, which can slow down the convergence process. GD can be slow, especially if the optimization landscape has many local minima, as it relies on the gradient to find the minimum.
2. Global Search Capability: PSO has a strong global search capability due to its ability to explore the search space more effectively by balancing exploration and exploitation. While GA also has good global search capabilities, it can sometimes get stuck in local optima due to premature convergence. GD is more prone to getting stuck in local minima, especially in non-convex optimization problems.
3. Ease of Implementation: PSO is relatively easy to implement and requires fewer parameters to be tuned compared to GA. GA require careful tuning of multiple parameters like population size, crossover rate, and mutation rate, which can be complex. GD requires the calculation of gradients, which can be computationally intensive and complex for certain functions.
4. Robustness to Noise: PSO is more robust to noise in the optimization landscape, making it suitable for real-world applications where the objective function may be noisy. GA can also handle noisy environments but may require more iterations to achieve convergence. GD can be sensitive to noise, which can affect the accuracy and stability of the optimization process.
5. Flexibility: PSO can be easily adapted and hybridized with other optimization techniques to improve performance further. GA are also flexible and can be combined with other methods, but the complexity of implementation increases. GD is less flexible in terms of hybridization with other algorithms.

Therefore, PSO is often preferred for beam-steering antenna optimization due to its faster convergence, strong global search capability, ease of implementation, robustness to noise, and flexibility. These advantages make PSO a powerful and efficient choice for optimizing complex antenna systems. The PSO approach optimizes the two lens’s position to maximize the produced NA gain G_o_ (θ^o^, φ^o^) while minimizing SLL, as described by the following objective function:

7 $$\:Objective\:function\:=max\:{G}_{o}\:\left({\theta\:}^{o}\:,\:{\phi\:}^{o}\:\right)\left|dBi\:+\:min\:{S}_{11}\right|dB\:\:,$$

whereas the antenna gain should not be less than 17 dBi with a minimum SLL of − 5 dB at least as convergence conditions. The gain of the investigated NAs is optimized for optical wireless applications at the typical telecommunication wavelength of 1550 nm (193.5 THz). In PSO, every solution in the method is represented by a point known as a particle in the search space^72,73^. As the particles travel around the D-dimensional issue space, they will all gain knowledge from each other’s most valuable experiences. The i-th particle’s position and velocity vectors are denoted, respectively, by $\:{X}_{i}=\left({x}_{i1},\:{x}_{i2},\:.,\:{x}_{iD}\right)\:$and$\:{\:V}_{i}=({v}_{i1},{v}_{i2},\:.,\:{v}_{iD})$, accordingly. To update the velocity matrix at each iteration t, each particle is required to be aware of its local and global optimal position vectors.$\:{\:Pbest}_{i}=\left({pbest}_{i1},\:{pbest}_{i2},\:\dots\:,\:{pbest}_{iD}\right)$ is the personal best position vector as represents the position at which each particle reached its maximum fitness value for the current iteration. The point in the solution space at which all particles attained their optimal fitness value is determined by the global best position vector $\:{Gbest}_{i}\:\left({gbest}_{1},\:{gbest}_{2},\:\dots\:,\:{gbest}_{D}\right).$ The following two dynamic equations can be used to calculate each particle’s velocity and new position:

8 $${v}_{id}^{t+1}=\:{v}_{id}^{t}+\:{c}_{1}{\:rand}_{1}\left({pbest}_{id}-\:{x}_{id}^{t}\right)+{c}_{2}{rand}_{2}\:\left({gbest}_{d}-\:{x}_{id}^{t}\right),$$

9 $$\:{x}_{id}^{t+1}=\:{x}_{id}^{t}+\:{v}_{id}^{t+1}\:\varDelta\:t,$$

where, the current acceleration constants, indicated by c_1_ and c_2_, show how the stochastic acceleration terms weigh each particle to move it towards the pbest and gbest places. Additionally, ∆t is used as a unit time step, rand_1_ and rand_2_ are two random values in the range [0, 1]. The PSO algorithm is used in this work, with swarm sizes of 74 iterations.

### Fabrication techniques for nanoantennas

The fabrication of nanoantennas involves several advanced techniques to achieve the precise geometries and properties required for their functionality^74–78^:

Electron Beam Lithography (EBL): EBL is a precise method for creating nanostructures by scanning a focused electron beam over a surface coated with an electron-sensitive resist. EBL Offers high resolution and flexibility in pattern design. Limitations include time and cost, making it unsuitable for large-scale production.

Focused Ion Beam (FIB) Milling: FIB uses a focused beam of ions to mill or deposit materials at the nanoscale. FIB provides real-time turnaround and high precision. Its drawbacks similar to EBL, it is slow and costly.

Nanoimprint Lithography (NIL): NIL involves pressing a mold with nanoscale features into a resist-coated substrate to create patterns. Its advantages include cost-effectiveness and fast throughput. Its limitations include challenges with mold manufacture and alignment.

Self-Assembly and Chemical Synthesis: Bottom-up approaches where nanoparticles self-assemble into desired structures. Although it offers less control over the final structure than top-down approaches, it has several benefits, including scalability and versatility.

#### Material limitations

Electrical Conductivity: Although highly plasmonic materials like gold and silver are frequently employed, they may experience significant resistive losses at optical frequencies. The solution is investigating substitute materials that have superior performance at the nanoscale, such as graphene or transition metal dichalcogenides (TMDs).

Dielectric Constant: The resonance frequency and effectiveness of nanoantennas are influenced by the dielectric characteristics of materials. Using materials with adjustable dielectric characteristics, like phase-change or metamaterials, is the answer.

Thermal Stability: The performance and longevity of nanoantennas can be impacted by severe heating. To control heat dissipation, the solution is to create materials with excellent thermal conductivity and stability.

#### Practical challenges in scaling for commercial or industrial applications

Manufacturing Scalability: Techniques like EBL and FIB are not suitable for mass production due to their high cost and low throughput. The solution is adopting high-throughput techniques like NIL or roll-to-roll processing for large-scale fabrication.

Material Uniformity: Ensuring consistent material properties across large batches of nanoantennas requires strict quality control procedures and the use of cutting-edge material synthesis methods.

Integration with Existing Technologies: Integrating nanoantennas with current electronic and photonic systems. To ensure compatibility and performance, the solution is creating hybrid systems that integrate nanoantennas with traditional components.

Cost-Effectiveness: Reducing the overall cost of nanoantenna production to make them viable for commercial applications. Solutions include streamlining fabrication processes and using cost-effective materials and techniques.

Consequently, while there are challenges in fabricating and scaling nanoantennas for commercial use, advancements in fabrication techniques, material science, and integration strategies hold promise for overcoming these obstacles. However, there are numerous published works introducing various fabricated nanoantennas.

Furthermore, from the perspective of lens antenna fabrication, 3D printing is a viable option to implement this technology in order to obtain permittivity values that are closer to those estimated. New printing materials, such as ceramics and low-loss ABS filaments, make it simple to fabricate dielectrics with varying relative permittivities and allow for any manufactured shape^52,79^, and^80^.

## Simulated results and discussion

The performance of the introduced hybrid plasmonic NA with two rectangular flat lenses is presented and discussed in this section, and it is contrasted with the literature review. Then, a representation of the beam-steering caused by the two lenses’ displacement will be provided. To maximize the gain in a specific direction, the two movable lenses are finally adjusted optimally using PSO.

### Hybrid plasmonic NA with two dielectric flat lenses

**Fig. 3:**
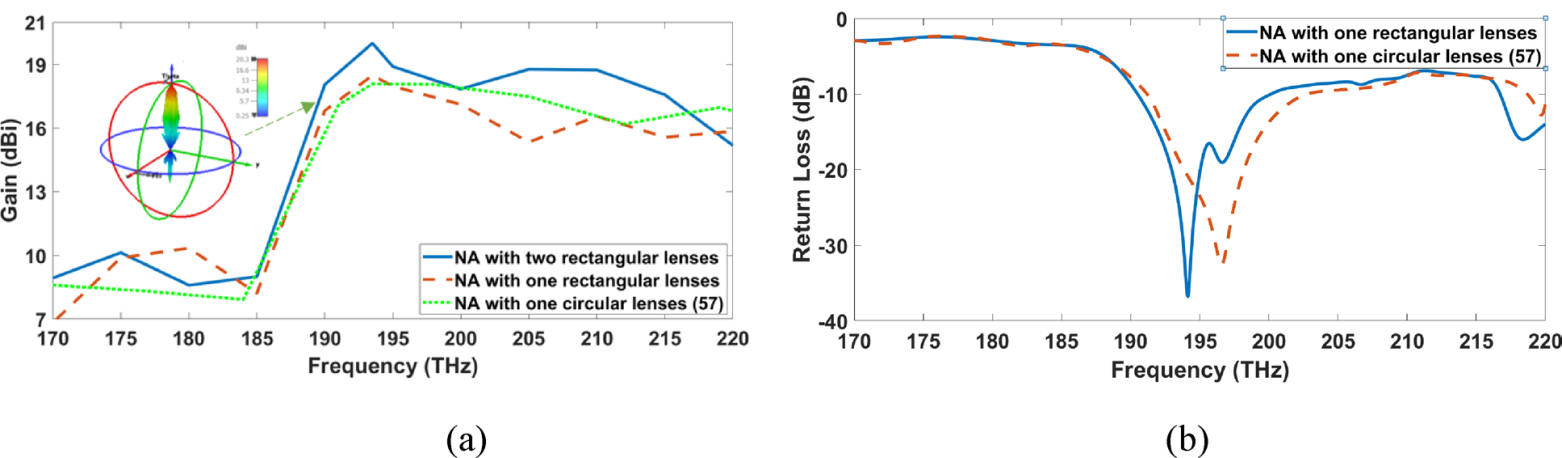
The radiation characteristics comparison of NAs with one or two flat lenses over the frequency range. (**a**) Gain, (**b**) return loss.

In Fig. 3a, the gain of the proposed elliptical patch plasmonic NA with a single rectangular flat lens and two lenses is shown and compared to the conventional NA with a circular lens^57^ along the frequency range (170–220) THz. The created elliptical patch NA with two rectangular lenses achieves the best gain of 20.3 dBi at λ = 1550 nm, which improves by 1.7 dB compared to the others with one lens. The NA with two rectangular lenses exhibits a 3-D radiation pattern that is included in Fig. 3a. The angles of azimuth and elevation are indicated by φ and θ, respectively. The main lobe is smooth, and vertically directed as illustrated in the Fig. 3a. However, additional side and back lobes of -9.7 dB and 10 dB are present because of the considerable back radiation caused by lens reflections. A half power beam-width (HPBW) of 9.4° has been determined. The gain of the NA with a rectangular and circular lens is 18.6 dBi and 18.4 dBi at 193.5 THz, respectively. It is observed that the rectangular lens has a simple gain enhancement, but it achieves good matching at the desired frequency when compared to the circular lens, as illustrated in Fig. 3b. The return loss of the NA with the rectangular and circular lenses was − 24.9 dB and − 19.15 dB, respectively, due to the rectangular lens’s large aperture and the low-loss substrate.

Side-lobe suppression is crucial in lens antenna design to minimize interference and improve signal quality. Here are some key techniques for side-lobe suppression, particularly when using multimaterial lenses. Multimaterial lenses, often created using advanced 3D printing techniques, offer unique advantages for side-lobe suppression^81^:

1. Material Variation:
  Gradient Index Lenses: By varying the refractive index across the lens, it is possible to control the phase and amplitude of the transmitted signals, reducing side-lobes.
  Layered Structures: Using different materials in a layered fashion can help in achieving desired electromagnetic properties, enhancing side-lobe suppression.
2. Customizable Designs:
  Tailored Optical Properties: Multimaterial lenses can be designed with specific optical properties to target and suppress side-lobes effectively.
  Complex Geometries: Advanced 3D printing allows for the creation of complex lens geometries that can better manage side-lobe levels and back radiation.

These techniques, combined with the flexibility of multimaterial lenses, provide a robust approach to addressing side-lobe levels and back radiation in antenna design.

The antenna performance compared with those in similar nano-antenna designs are shown in Table 1. It is noticed that the NA with two rectangular lens is the highest gain of 20.3 dBi and the best HPBW of 9.4° from NA in the literature reviews^57^ and NA with single circular or rectangular lens. However, side lobe level of the NA with circular lens is the best reach to − 12.3 dB and return loss of the NA with rectangular lens is the best reach to − 24.9 dB. NAs with two rectangular lenses are preferred due to the trade-offs between their antenna performance.

**Table 1:** The radiation characteristic comparison for different NAs at 1550 nm.

	NA without lens (57)	NA with circular lens	NA with rectangular lens	NA with two rectangular lens
Gain (dBi)	10.7	18.6	18.4	20.3
Return loss (dB)	− 14.41	− 19.15	− 24.9	− 19.82
SLL (dB)	− 2.7	− 12.3	− 9.6	− 9.7
HPBW (°)	30.2	13.6	15.7	9.4

Studying the performance of lens antenna setups is essential for optimizing efficiency, enhancing aperture efficiency, minimizing scan loss^82–85^. Efficiency in lens antennas refers to how effectively the antenna converts input power into radiated power. High-quality materials with low absorption and scattering improve efficiency. Optimized shapes and coatings reduce losses and enhance transmission. Designs such as graded index lenses can significantly improve efficiency by minimizing phase errors. Precise alignment of lenses ensures minimal loss and maximum throughput. Misalignment can lead to significant efficiency losses. The efficiency of the two lens setup is -0.2658 dB equivalent to approximately 93.9%. Aperture efficiency measures how well the lens system utilizes the available aperture to focus energy. It is defined as the ratio of the effective radiating area to the physical area of the aperture. High aperture efficiency indicates that most of the aperture contributes to the radiation, leading to better performance. Larger apertures generally provide better efficiency but must minimize aberrations. Affects beam width and concentration, impacting aperture efficiency. Properly designed edges and coatings reduce diffraction and improve efficiency. Techniques like phase smoothing compensation can enhance aperture efficiency. The two lens setup has an aperture efficiency of -0.2203 dB, which is corresponding to approximately 95.1%. Scan loss refers to the reduction in signal strength when the beam is steered away from the central axis. Mechanical systems may have lower scan loss compared to electronic systems due to fewer phase errors. However, mechanical systems can be slower. Optimized designs can minimize scan loss by maintaining beam integrity over a wide range of angles. Higher frequencies may experience greater scan loss due to increased sensitivity to alignment and material properties. Proper design can mitigate these losses. The two lens setup has scan loss of 2.4 dB for a scan angle of ± 40° along y-axis and 3.6 dB for a scan angle of ± 45° along x-axis. Typically, scan loss increases with the scan angle, leading to reduced gain and efficiency at larger angles.

**Fig. 4:**
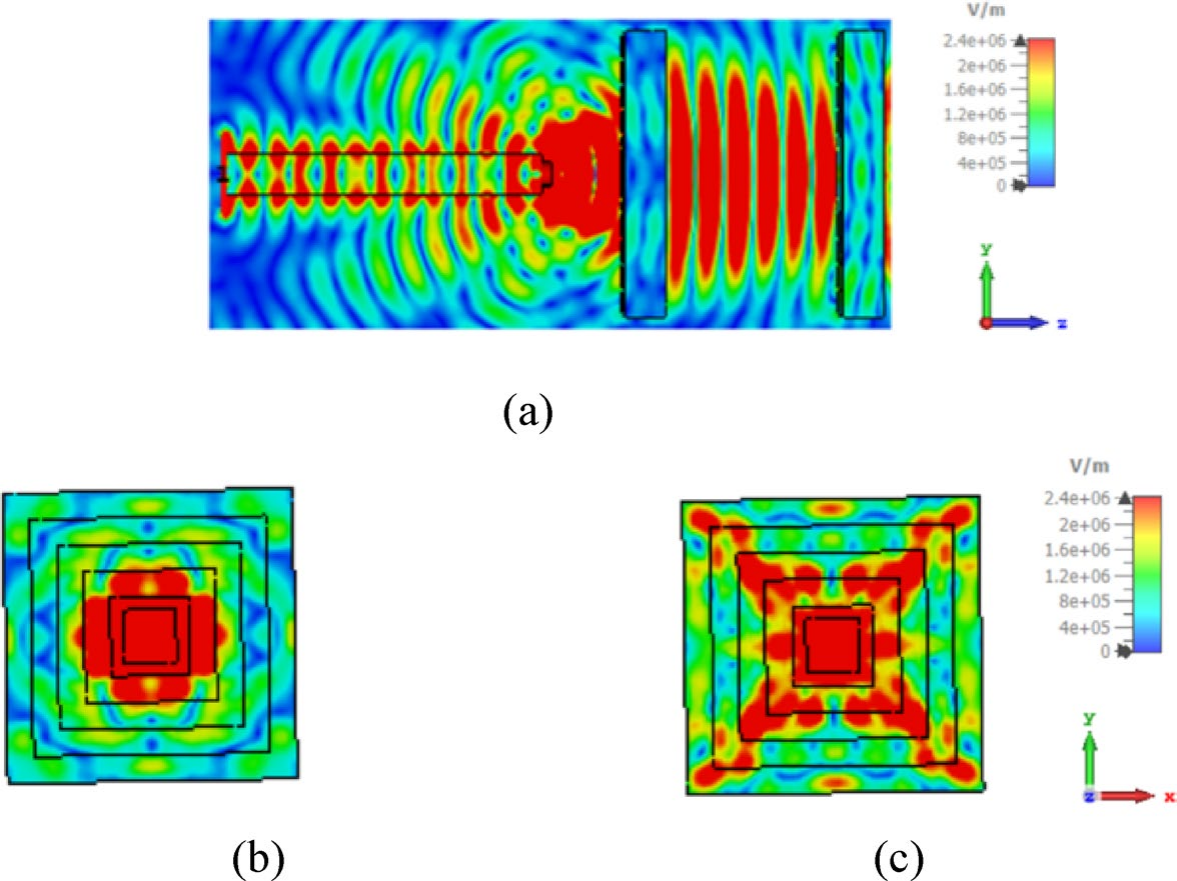
Simulated electric field at 1550 nm in the introduced NA with two lenses for (**a**) a side view, and (**b**, and **c**) the apertures of the lower and higher lenses, respectively.

Figure 4a shows a side view of the simulated electric field at 1550 nm in the introduced NA with two rectangular flat lenses. However, the amplitude aperture distribution is taken from the surface plane in front of each lens aperture, as illustrated in Fig. 4b, c, where the near intensity of the lower and higher lenses are presented in Fig. 4b, c, respectively. The simulation findings show that the two rectangular lenses that were created have low backscatter characteristics and perform effectively in converting the incident wavefront into a plane wave.

### Beam-steering based on two transferring lenses

**Fig. 5:**
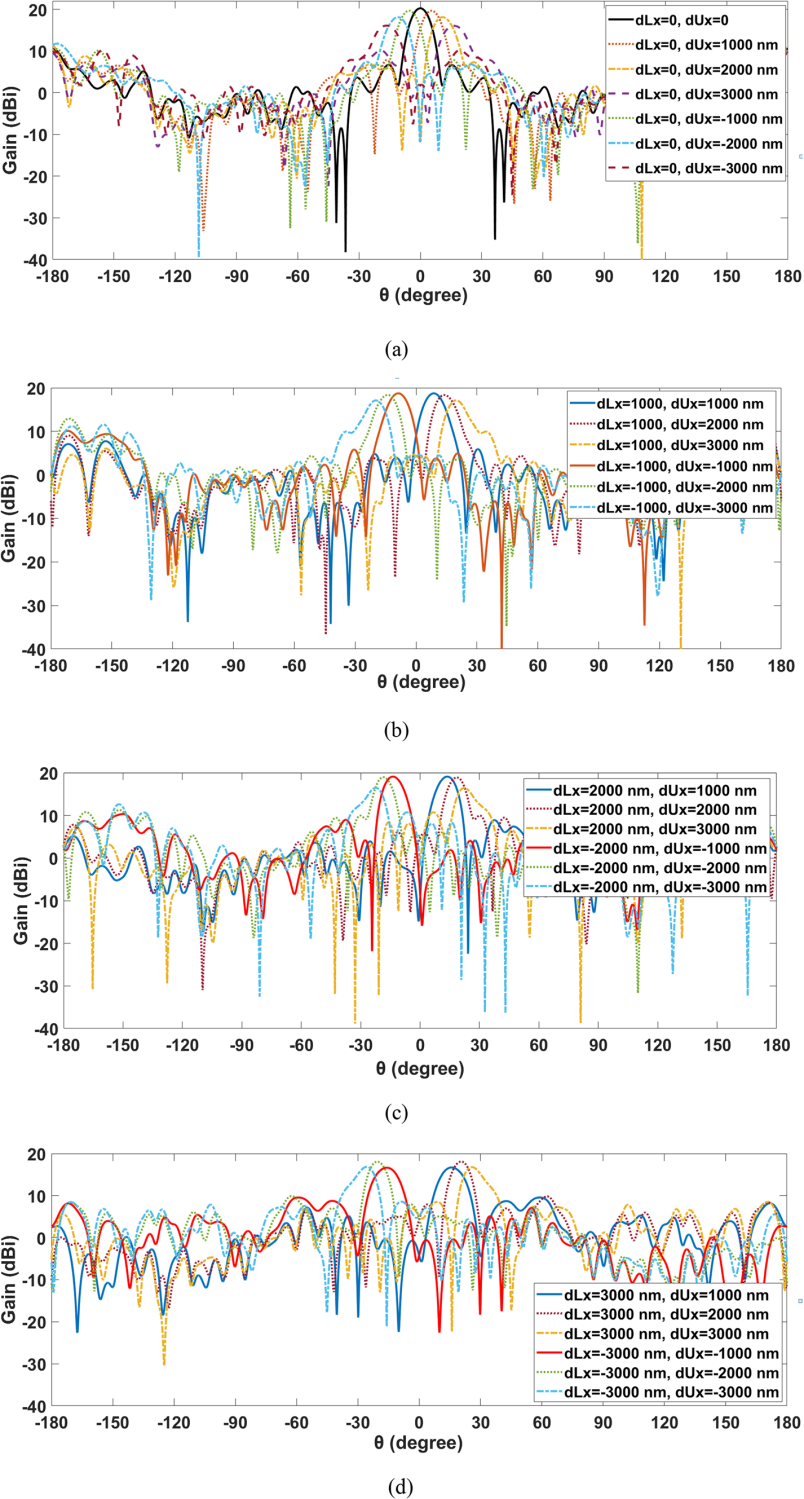
2-D steering radiation pattern of the produced NA with two movable lenses in the x-z plane at 1550 nm.

In this part, the beam steerable NA by two shifting flat lenses is presented, whereas, beam-steering will be achieved by displacing each of the two lenses. Several displacements of each of the lenses have been performed corresponding to various positions along the X and Y axes. The lower lens is the nearer lens and is displaced in different positions in X and Y directions and defined by dLx and dLy, respectively. The upper lens is the farthest and is displaced in different positions in X and Y directions by dUx and dUy, accordingly. Figure 5 shows the beam-steering capabilities in the x–z plane (φ = 0°). Figure 5a shows the capabilities of beam-steering when the lower lens is constant at x = 0 and the upper lens is transferring along X-directions. Additionally, beam-steering skills are seen when the lower lens is shifting by dLx = 1000 nm in the positive or negative directions and stopped while the upper lens is transferring along X-directions as shown in Fig. 5b. Similarly, Fig. 5c, d demonstrate when the lower lenses ceased moving at dLx= (2000 and 3000 nm) in the positive and negative directions while the upper lens moved along the x-axis at dUx= (1000 nm, 2000 nm, and 3000 nm). Table 2 depicts test scenarios that demonstrate the effect of different sites on the movement of the lower and upper lenses at the X-axis in the radiation parameters of the main lobe.

**Table 2:** The antenna performance at various positions of lower and upper lenses’ displacement along the x-axis.

Lower lens displacement dLx (nm)	Upper lens displacement dUx (nm)	Gain (dBi)	Return loss (dB)	HPBW (°)	SLL (dB)
0	0	20.3	− 19.82	9.4	− 9.7
0	± 1000	19.6	− 15.4	9.4	− 9.2
	± 2000	18	− 14	10.3	− 6.3
	± 3000	15.9	− 14	12.3	− 5.9
± 1000	± 1000	18.6	− 19.5	10.4	− 8.5
	± 2000	18.2	− 20.8	10.4	− 5.6
	± 3000	17.1	− 19.3	10.2	− 5.6
± 2000	± 1000	18.9	− 17.75	10.3	− 8.8
	± 2000	18.6	− 17.03	9.2	− 7.6
	± 3000	15.7	− 16.5	10.3	− 4.3
± 3000	± 1000	16.7	− 17.45	12.7	− 7.1
	± 2000	18.1	− 18.3	9.3	− 8.3
	± 3000	16.7	− 18.86	10.7	− 8.3

**Fig. 6:**
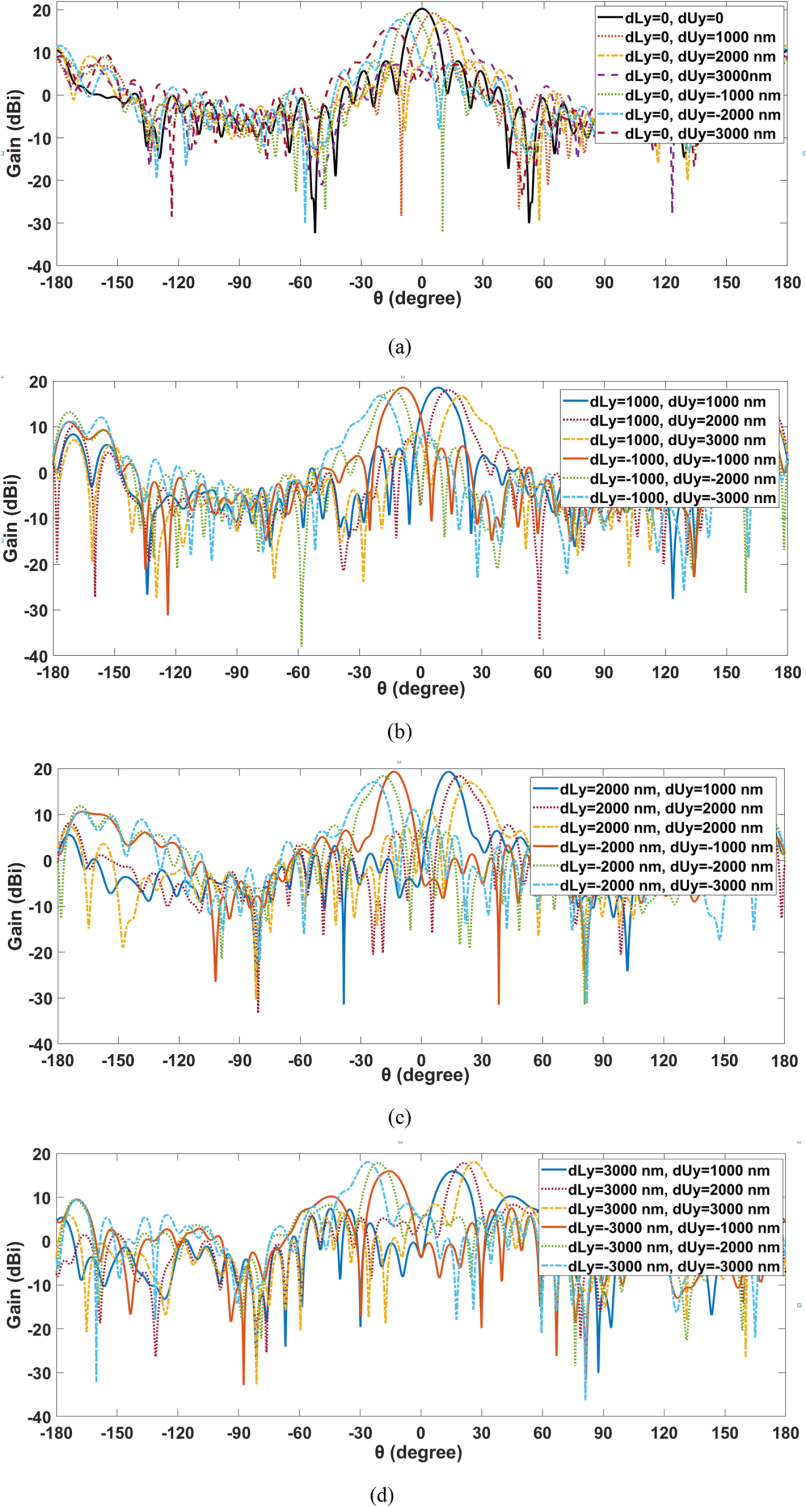
2-D steering radiation pattern of the produced NA with two movable lenses in the y–z plane.

Likewise, The beam-steering capabilities in the y-z plane (φ = 90°) are displayed in Fig. 6. When the upper lens transfers along y-directions while the bottom lens remains constant at y = 0, Fig. 6a illustrates the beam-steering abilities. Figure 6b illustrates the beam-steering skills when the upper lens is transferring along y-directions while the bottom lens is shifting by dLy = 1000 nm in both a positive or negative direction and stopped. The upper lens moved along the x-axis at dUy= (1000 nm, 2000 nm, and 3000 nm), while the lower lenses stopped moving at dLy= (2000 and 3000 nm) in the positive and negative directions are shown in Fig. 6c, d. Additionally, scenario tests showing the impact of various sites of the movement of the upper and lower lenses in the y-axis in the radiation characteristics of the major lobe are shown in Table 3.

It is observed that by mechanically altering one or both lenses’ displacement, the aperture phase distribution can be changed, enabling continuous beam guidance in the azimuthal plane at a wide angle. It is demonstrated that beam-steering performance declines when the locations of two lenses are altered from their initial configuration. It is evident that the beams exhibit spatial differentiation as they pass through the lens, resulting in a continuous beam-steering range of approximately ± 45° × ±40°. Along with preserving a low side lobe level of -4.3 dB and an acceptable gain value of at least 15.7 dBi over a range of steering angles, the HPBW fluctuates around 10° at 193.5 THz. The proposed system provided acceptable beam-steering characteristics while using one antenna element and simple mechanical motion to two movable lenses, as opposed to an antenna array approach.

**Table 3:** Antenna performance at different locations of lower and upper lens displacement along the y-axis.

Lower lens displacement dLy (nm)	Upper lens displacement dUy (nm)	Gain (dBi)	Return loss (dB)	HPBW (°)	SLL (dB)
0	0	20.3	− 19.82	9.4	− 9.7
0	± 1000	19.2	− 16.5	9.3	− 9.9
	± 2000	17.7	− 14	10.4	− 6.7
	± 3000	15.7	− 13.7	10.6	− 6.7
± 1000	± 1000	18.4	− 21	10.6	− 6.2
	± 2000	17.9	− 20	10.6	− 5.7
	± 3000	16.8	− 19	10.4	− 7.8
± 2000	± 1000	19	− 17.8	10.3	− 9
	± 2000	18.2	− 17.4	9.8	− 7
	± 3000	16.9	− 16.6	10.5	− 6.5
± 3000	± 1000	15.9	− 18.5	12.5	− 5.9
	± 2000	17.7	− 18.7	9.5	− 7.8
	± 3000	17.9	− 18.4	9.8	− 7

Beam-steering occurs when the upper and lower lenses’ positions are displaced along the x and y axes. As the displacement of the two lenses increases, the beam form in the main direction degrades, as illustrated in Figs. 5a–d and 6a–d. Furthermore, beam-steering with Y-displacement has higher gain than beam-steering with X-displacement. Additionally, the SLL degradation of the introduced NA with two lens displacements along the x-axis is less than that of the NA with two lens displacements along the y-axis.

As the steering range of electronic and phase array systems increases, the complexity of the control electronics and the overall system architecture can also increase^86^. This added complexity can lead to higher costs and potential reliability issues. At the edges of the steering range, maintaining beam quality and precision can require significantly more power. This is due to the increased effort needed to control the beam direction accurately over a wider range. High power consumption can lead to thermal issues, affecting the system’s performance and longevity.

These trade-offs highlight the importance of carefully balancing various factors to optimize the performance of beam steering systems, especially at the edges of their steering range. At the edge of the steering range at ± 45° along the x-axis, the introduced NA reach up to gain of 16.7 dBi, and SLL equal to -8.3 dB when each of the two lenses shifted by ± 3000 nm. Furthermore, when both lenses were displaced by ± 3000 nm at ± 40° along the y axis, the produced NA reached a gain of 17.9 dBi at the edge of the steering range, and the SLL was equal to -7 dB.

**Fig. 7:**
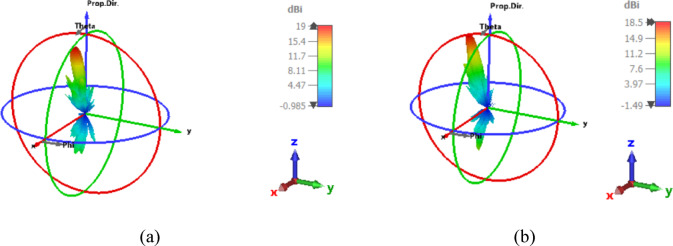
3-D radiation patterns of the optimized positions of the two lenses displacement a specific direction at 1550 nm (**a**) at X-direction (**b**) at Y-direction.

Figure 7 represents the 3-D radiation patterns of beam-steering utilizing PSO to optimize the two lenses’ placements to maximize antenna gain in the targeted location. As demonstrated in Fig. 7a, the provided NA causes positive displacement of lenses in the X direction at 1550 nm. Using PSO, an optimal gain of 19 dBi is attained in the desired direction of 20 degrees, with a low loss of − 18.07 dB, a side lobe level of -9.3 dB, and a half power beam-width of 9° at 193.5 THz. Furthermore, Fig. 7b depicts the 3-D radiation patterns of the lenses’ displacement in the Y-axis towards the negative direction. The targeted angle of 30 degrees is achieved with an optimal gain of 18.5 dBi, low loss of -18.18 dB, side lobe level of -10 dB, and HPBW equal to 9°. The optimized parameters of (dLx, and dUx) in X-motion and (dLy, and dUy) in Y-motion are listed in Table 4 with swarm sizes of 74 iterations. It is found that the optimized displacement shows optimal radiation parameters in the forward direction.

**Table 4:** The optimized parameters of lower and upper lenses’ displacement.

Displacement direction	Parameter	Decision space (nm)		Optimized results (nm)
		From	To	
X-displacement	dLx	2000	3000	2407.1
	dUx	1000	2000	1646.62
Y-displacement	dLy	− 1000	− 2000	− 1047
	dUy	− 1000	− 2000	− 1673.4

## Conclusion

In this paper, a dual-lens hybrid plasmonic NA design is displayed. Electromagnetic full-wave simulations are used to analyze this device’s performance, and a numerical assessment of the entire system is conducted. A typical elliptical patch NA without lenses yields a return loss of -14.41 dB and a gain of up to 10.7 dBi. To improve gain while minimizing beam width, the design with two gradient-index dielectric flat lenses is introduced to improve antenna performance. Additionally, the two lenses’ displacement by the various lens positions along the X and Y axes provides beam-steering capabilities. In comparison to standard NA, the gain is boosted to 20.3 dBi and the return loss is improved to -24.9 dB by employing the two gradient-index dielectric flat lenses. Furthermore, a range of ± 45° × ±40° was attained for the beam-steering capabilities, with an acceptable average antenna gain, side-lobe levels, and half power beam-width of 18 dBi, − 7.4 dB, and 10.7°, respectively. Moreover, the PSO method is proposed to maximize the pre-specified fitness function.

## Methods

To analyze the performance of the entire structure, a 3D full-wave numerical simulation was performed, and the Uni-directional simulation setup, with the concept design placed into the SiO_2_ background and the boundary conditions defined as open-add-space (modelling the radiation condition). The simulation was run in two stages. The inserted nanoantennas were analyzed using the Finite Elements method during the first stage. Step two involved illuminating the lenses with the findings of step one and displacement of the nanoantenna to achieve beam-steering.

## Data Availability

The datasets used and/or analysed during the current investigation are available upon reasonable request from the corresponding author.
